# Supplementary material for: Generalization properties of neural network approximations to frustrated magnet ground states
Source: Nat Commun. 2020 Mar 27;11:1593. doi: 10.1038/s41467-020-15402-w (PMC7101385; doi:10.1038/s41467-020-15402-w)
Supplement: Supplementary file 1 — Supplementary Information [file 41467_2020_15402_MOESM1_ESM.pdf]

Supplementary Information

to the article "Generalization properties of neural network approximations to frustrated magnet ground states" by T. Westerhout et al.

In this Supplemental Information, we provide some additional details regarding the numerical analysis outlined in the main part of the paper.

SUPPLEMENTARY NOTE 1: NETWORK ARCHITECTURES

We use three different Neural Network architectures to demonstrate that the effects we observe might be general for all standard deep learning techniques.

a. One-layer dense network can be applied to all three models at hand. We arrange the spins in a linear pattern and feed them to the network which has one hidden layer of 64 neurons and the `ReLU` non-linearity. When it is used to approximate wave function amplitudes, only one output representing logarithm of the amplitude is taken. When it is used to learn the sign structure, it is equipped with two output nodes representing non-normalized probabilities of the sign to be ± 1. Then, when the standard PyTorch cross entropy loss function is applied, those are converted into a single number $p \in [0, 1]$ with `SoftMax`. The total number of parameters in this network is $N \times 64 + 64 + 64 \times 2 = 1728$ (for 24 spins) or 2112 (for 30 spins).

b. Two-layer dense network can be applied to all three models at hand as well, and has a very similar structure as the one-layer one. Both hidden layers have 64 neurons. `ReLU` non-linearity is applied after every hidden layer. The total number of parameters is $N \times 64 + 64 + 64 \times 64 + 64 + 64 \times 2 = 5888$ (for 24 spins) or 6272 (for 30 spins).

c. Periodic convolutional network can applied to the J_1–J_2 model on a square lattice. The main goal of this architecture is to preserve the translational invariance on the level of NN architecture. In every convolutional layer we apply periodic boundary conditions. When all the convolutional layers are applied, we take mean value operation over all spins in every "channel", i.e. over all lattice sites. One can check that the prediction of such neural network is invariant with respect to any translation of the input spin configuration.

For the convolutional NN (CNN) architecture, we provide a listing of Python code using the PyTorch NN manipulation package. In what follows, the input parameter `is_odd` allows for selection between the momenta 0 and π in the y-direction. The goal of this architecture is to "hard-code" the translational invariance, i.e. if two outputs of the neural network mean probabilities of "+" and "−" signs, we would like them to remain unchanged in "even" case ($p_y = 0$) and exchange in the "odd" case ($p_y = \pi$) upon shift of the spin configuration in y direction. In order to achieve this property, the following procedure is performed. Before the action of every convolutional layer we periodically pad the input such that the valid convolution (convolving only the actual elements without going beyond the boundary) shrinks the configuration back to its initial size `x_size`×`y_size`. After applying all the convolutional layers we perform weighted averaging over two spatial dimensions. All weights have the same absolute value and are all positive in the "even" case and have alternating signs in the "odd" case. The resulting vector of the length `n_channels` is mapped into one single number r by the dense layer without bias. This number has the property that it changes sign when the configuration is shifted in y-direction in the "odd" case and remains unchanged in the "even" case. It is then mapped onto two probabilities as

$$[p, 1-p] = \left[\frac{1+\tanh r}{2}, \frac{1-\tanh r}{2}\right]$$

which are then used to infer the sign of the configuration. It can be easily seen that the resulting probabilities are exchanged (and the sign is flipped in the "odd" case and remain intact in the "even" case upon the shift in y-direction), as required.

```python
import torch

# 2D convolution preserving translational symmetry
class Conv2d(torch.nn.Module):
    def __init__(self, *args, **kwargs):
        super().__init__()
        self.conv = torch.nn.Conv2d(*args, **kwargs)

    def forward(self, x):
        # Periodic padding
        x = torch.cat([x, x[:, :, : self.conv.kernel_size[0] - 1, :]], dim=2)
        x = torch.cat([x, x[:, :, :, : self.conv.kernel_size[1] - 1]], dim=3)
        # Convolution + activation
        x = torch.relu(self.conv(x))
        return x

# Neural network predicting the sign structure.
class Net(torch.nn.Module):
    __constants__ = ["is_odd"]

    def __init__(self, is_odd=False):
        super().__init__()
        # Two convolutional layers
        self.conv1 = Conv2d(1, 32, kernel_size=5)
        self.conv2 = Conv2d(32, 64, kernel_size=5)
        # Final dense layer
        self.dense = torch.nn.Linear(64, 1, bias=False)

    def forward(self, x):
        x = x.view((x.size(0), 1) + self.size()[1:])
        # Convolutions
        x = self.conv1(x)
        x = self.conv2(x)
        # Enforcing translational symmetry by weighted averaging
        if self.is_odd:
            x[:, :, ::2, :] *= -1
        x = x.mean(dim=(2, 3))
        # Final dense layer
        x = 0.5 * (1 + torch.tanh(self.dense(x)))
        # Returning probabilities
        return torch.cat([x, 1 - x], dim=1)
```

SUPPLEMENTARY NOTE 2: RESULTS OF ADDITIONAL NUMERICAL EXPERIMENTS

A. 24 spins

In this section, we provide some additional results for frustrated 24-spin clusters supporting the statements which we made in the main part of the paper. These results were obtained with two-layer dense network described in Supplementary Note 1.

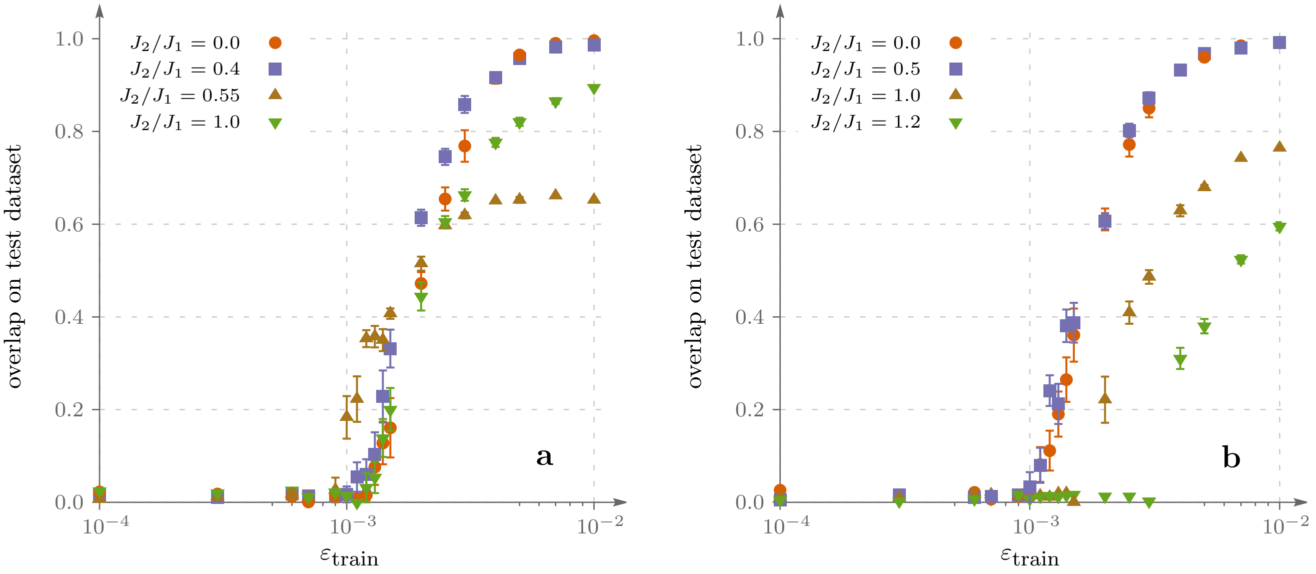

Supplementary Figure 1. Dependence of sign structure generalization quality on the size of the training dataset. We show results for square (**a**) and triangular (**b**) lattices (results for Kagome lattice were presented in the main text).

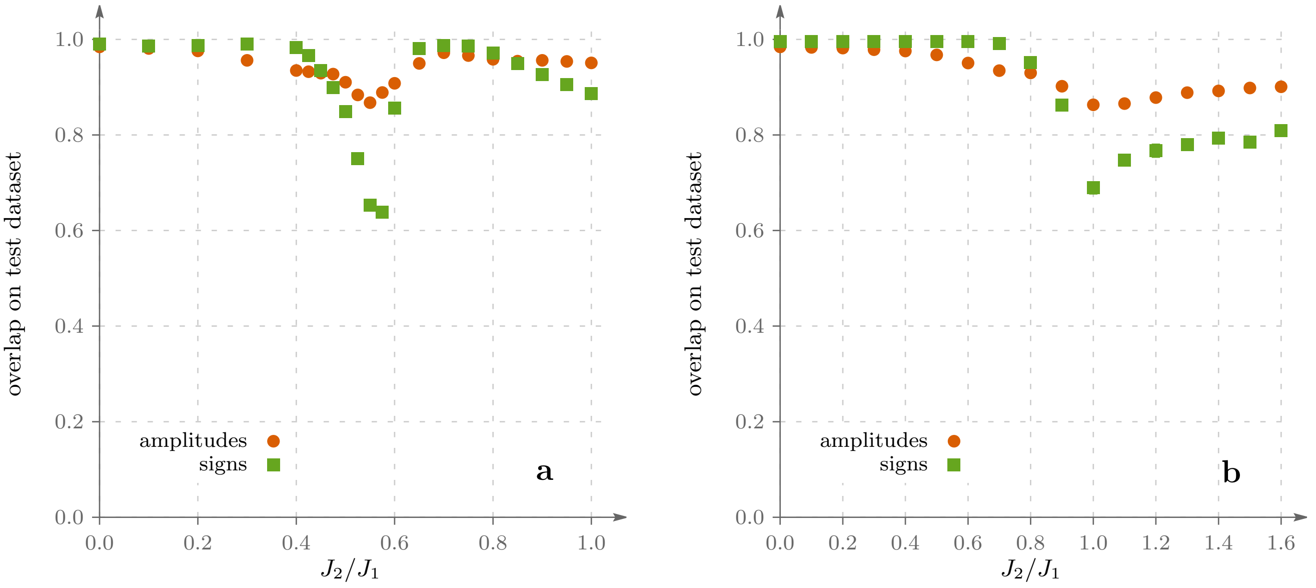

Supplementary Figure 2. Generalization quality of signs and amplitudes. We compare generalization quality for learning the sign structure (green squares) and amplitude structure (orange circles) for the square (**a**) and triangular (**b**) lattices. Results for Kagome lattice were presented in the main text

In Supplementary Figure 1, we show the dependence of sign structure generalization quality on the training dataset size. We consider two lattices: square (left) and triangular (right). In both lattices, generalization quality exhibits a

sharp transition around certain critical value of $\varepsilon_{\text{train}}$. This is qualitatively the same as what we have observed for the Kagome lattice in the main text. It is interesting to note that for the square lattice, the transition occurs at the same $\varepsilon^*_{\text{train}}$ for different values of J_2/J_1, while for the triangular and Kagome lattices $\varepsilon^*_{\text{train}}$ increases upon increasing the frustration.

In Supplementary Figure 2, we again show results for square and triangular lattices and highlight how difficult it is to generalize the sign structure compared to amplitude structure. Although for both learning tasks there is a dip in the generalization quality, it is evident that the sign structure is more difficult to learn in the frustrated regime.

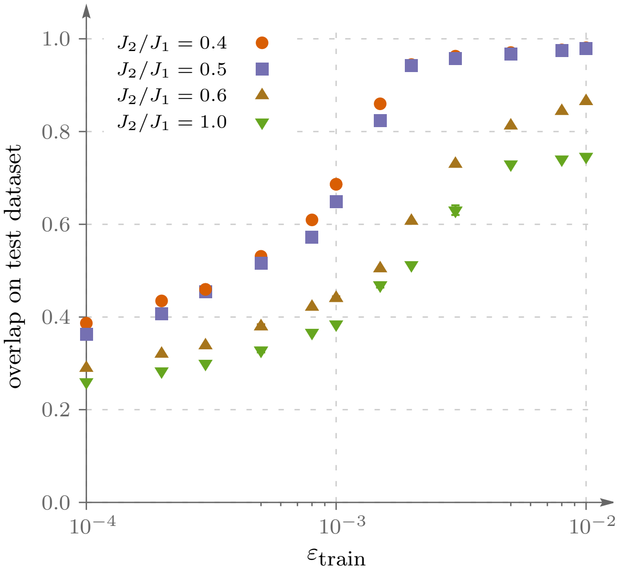

Supplementary Figure 3. Dependence of the amplitude generalization quality on the training dataset size for Kagome lattice. Note that no step-like behavior is observed.

In Supplementary Figure 3, we show results for amplitude generalization quality dependence on $\varepsilon_{\text{train}}$, demonstrating that it exhibits smooth behavior in contrast with the one for the sign structure.

B. 30 spins

In this section, for the clusters of 30 spins we reproduce all the results that we obtained in the main text for 24-spin systems to demonstrate that our conclusions are valid for larger systems.

In Supplementary Figure 4, we plot the dependence of generalization quality on J_2/J_1. Clearly, the patterns are very similar to what we have observed for clusters of 24 spins.

In Supplementary Figure 5, the abrupt improvement of generalization quality at some critical training dataset size $\varepsilon^*_{\text{train}}$ is shown. Comparing that to Fig. 3 in the main text, one can see that for larger systems a considerably smaller fraction of basis vectors is required for NN to start properly generalize the sign structure.

In Supplementary Figure 6, using the 2-layer dense neural network, we show that in the frustrated regime signs are more difficult to generalize than amplitudes.

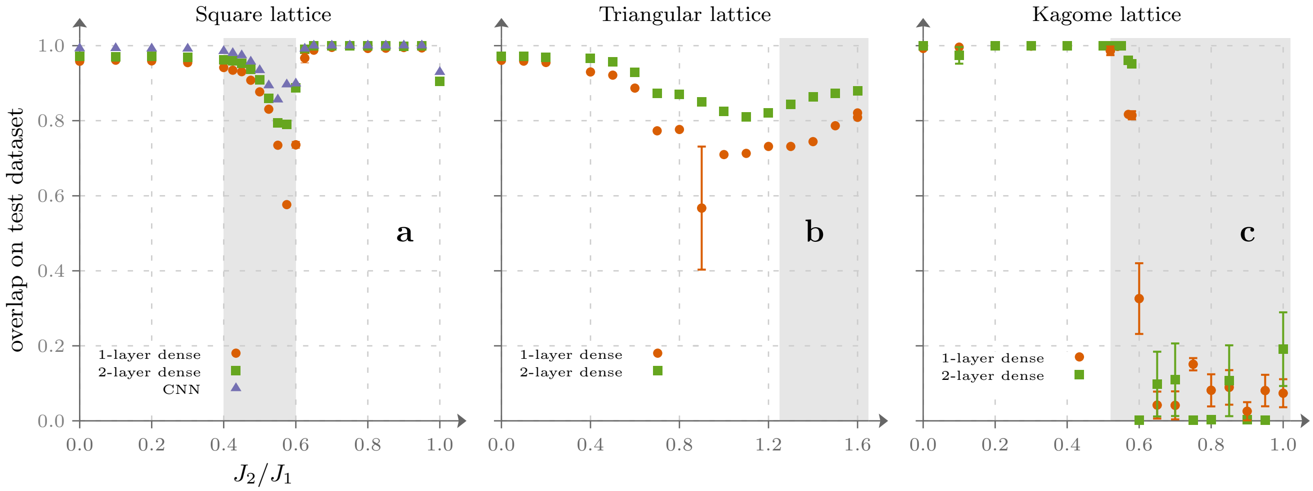

Supplementary Figure 4. Dependence of sign structure generalization quality on frustration level. We show overlap (computed on the test dataset) of the variational wave function with the exact ground state for square (**a**), triangular (**b**), and Kagome (**c**) lattices.

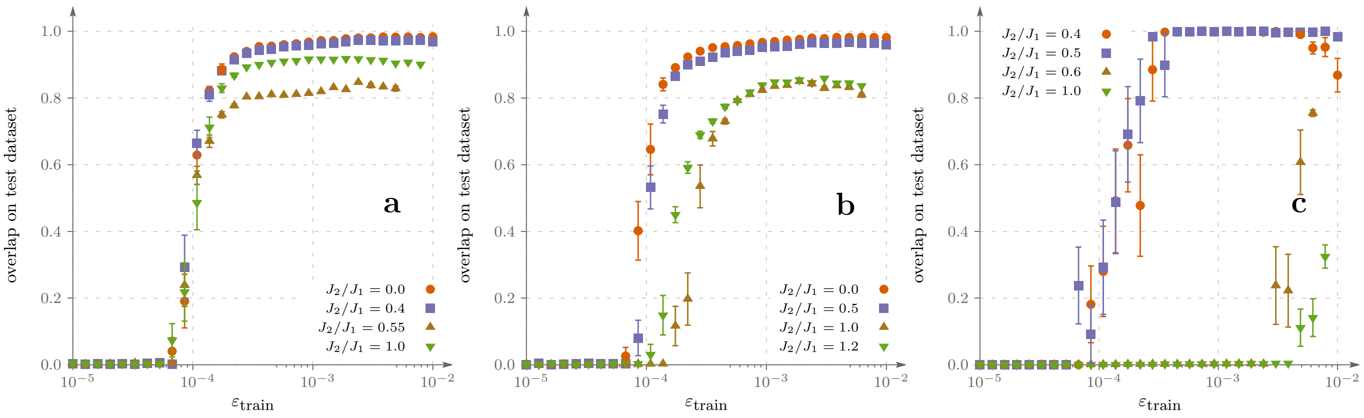

Supplementary Figure 5. Dependence of sign structure generalization quality on training dataset size. Results are shown for three lattices: square (**a**), triangular (**b**), and Kagome (**c**).

C. 32 & 36 spins

To estimate scaling of the critical training dataset size with system size (Fig. 4 of the main text), we have calculated $\varepsilon_{\text{train}}$–dependence of the sign structure generalization quality for the square lattice for even bigger systems. Supplementary Figure 7 shows results for 32- and 36-spin clusters.

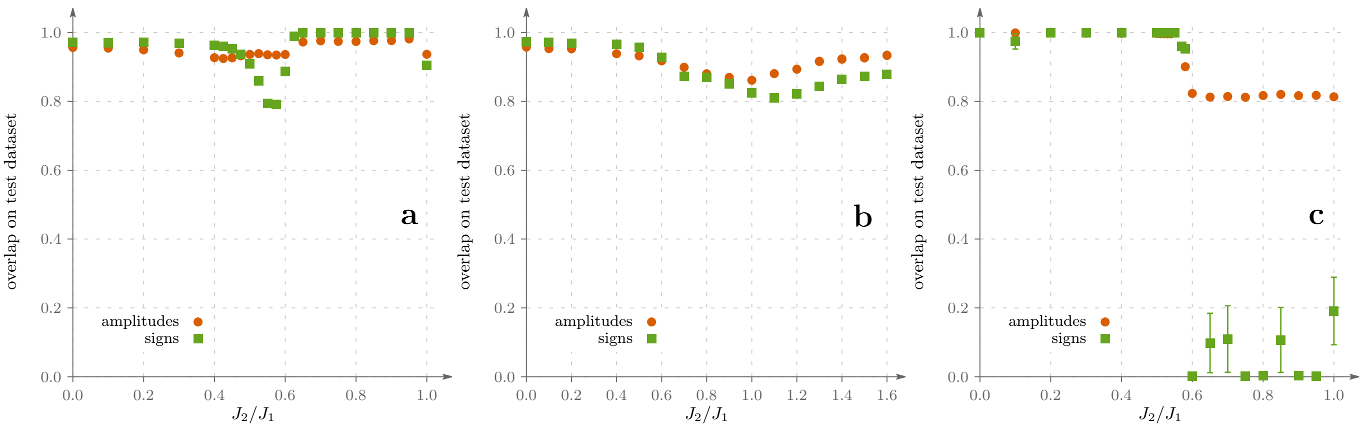

Supplementary Figure 6. Generalization quality of signs and amplitudes. We show generalization quality for learning the sign structure (green squares) and amplitude structure (orange circles) for the J_1–J_2 model on the square (**a**), triangular (**b**), and Kagome (**c**) lattices with 2-layer dense architecture employed both for signs and amplitudes.

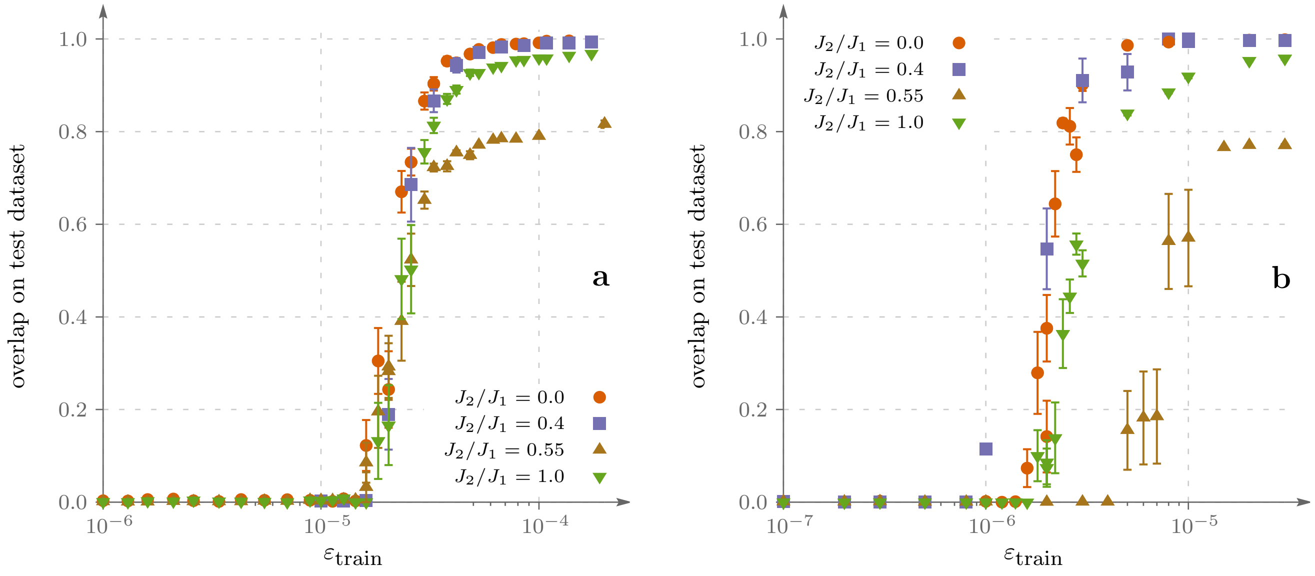

Supplementary Figure 7. Dependence of sign structure generalization quality on the size of the training dataset. $\varepsilon_{\text{train}}$ denotes the fraction of Hilbert space basis used for training. We show results for J_1–J_2 antiferromagnetic Heisenberg model on square lattice with 32 (**a**) and 36 (**b**) spins.
